# Neurological and Psychiatric Disorders in the COVID-19 Era

**DOI:** 10.3390/brainsci14040355

**Published:** 2024-04-02

**Authors:** Tommaso Ercoli, Francesco Loy, Carla Masala, Paolo Solla

**Affiliations:** 1 Department of Neurology, University of Sassari, Viale S. Pietro 10, 07100 Sassari, Italy; psolla@uniss.it; 2 Department of Biomedical Sciences, University of Cagliari, SP8 Cittadella Universitaria Monserrato, 09042 Cagliari, Italy; floy@unica.it (F.L.); cmasala@unica.it (C.M.)

Over the 4 last years, severe acute respiratory syndrome coronavirus 2 (SARS-CoV-2) has determined the diffusion of the coronavirus disease 2019 (COVID-19) global outbreak [1]. Neurological and psychiatric disorders have been described in patients both during the acute phase of COVID-19 and as long-term sequalae [2]. Common neurological or psychiatric symptoms during COVID-19 were, for instance, olfactory/taste dysfunction, headache, and anxiety. Researchers from all over the world have also focused their attention on the possible long-term symptoms that may persist after recovery from COVID-19 [3]. Several studies have described patients with permanent neuropsychiatric symptoms, including mood disorders, anxiety, and sensory disturbance [4]. Due to local restrictions and the increased number of patients requiring specific medical assistance, the management of neurological and psychiatric patients underwent profound changes in both outpatient and inpatient settings [5].

Within this context, in this Special Issue entitled “Neurological and Psychiatric Disorders in the COVID-19 Era”, we aimed to expand the current knowledge about neurological and psychiatric disorders in the COVID-19 period, and how the outbreak has impacted the management of these disorders. For this purpose, we provide an overview of the six original high-quality scientific papers included in the aforementioned Special Issue collection.

In the first study, Bakhsh and colleagues [6] performed a study on 63 individuals with COVID-19, aiming to determine the prevalence of pulmonary embolism in COVID-19 patients and the relationship between pulmonary embolism and long-term neurological sequalae. Interestingly, the authors found an association between pulmonary embolism in COVID-19 patients and neurological complications, with particular reference to stroke, seizures, migraine, and peripheral neuropathy. Therefore, this study highlighted the need for a careful neurological assessment in patients with COVID-19 suffering from pulmonary embolism in order to rapidly detect possible complications [6].

In the second study, Askari and colleagues provided a narrative review on the possible mechanisms of damage of SARS-CoV-2 in the whole body [7]. The paper covered an exhaustive section about neurological disorders, which included potential SARS-CoV-2-mediated brain injury mechanisms and COVID-19-associated neurological symptoms (i.e., cerebrovascular diseases, encephalitis, seizures, Guillain–Barré Syndrome, and neurodegenerative and demyelinating disorders) [7].

The third study by Camargo and colleagues [8] presents a systematic review that included 53 papers studying the possible factors that might determine or worsen depressive symptoms during COVID-19-related restrictions. Female sex, low educational level, young age, economic issues, comorbidities, and a history of previous depressive episodes were considered the most significant risk factors associated with the development of depression. Since there were several discrepancies between studies, the authors stressed the need for new research efforts that might assess the impact of confinement measures on mental health [8].

In the fourth study, Colizzi and colleagues [9] evaluated long-term neuropsychiatric sequalae in 230 patients over a 2-year period following COVID-19 infection. The authors reported that 36.1% of their cohort still had one long-lasting symptom from the first infection. Dyspnea at onset was indicated as a risk factor of the development of both psychiatric disorders and a lack of concentration/focus 24 months post infection. These findings might have clinical and direct implications, since the COVID-19 survivors seemed to be still in need of neuropsychiatric support [9].

The fifth study by Ercoli and colleagues [10] is a systematic review about the impact of COVID-19 on neurology training. Over recent years, neurology residents have experienced drastic changes in their clinical practice [11]. The authors covered several aspects including clinical and research activities, the implementation and use of telemedicine, the delivery of education, and the psychological well-being of the residents. The lessons learned during the COVID-19 outbreak might ensure that residents receive effective training even during this kind of crisis [10].

In the last paper published in this Special Issue, Abbruzzese and colleagues [12] evaluated emotional distress in 40 patients with COVID-19 admitted to a rehabilitation unit after discharge from intensive care. Interestingly, they did not find any significant differences between patients who underwent invasive ventilation and those who did not [12].

In conclusion, this Special Issue has collected different relevant studies that might help to better understand the intricate relationship between COVID-19 and neurological and psychiatric disorders. The ultimate goal of this Special Issue was to prove new hints for future research initiatives. As Guest Editors, we would like to thank all the authors and reviewers that have participated to this project, and the editorial team of Brain Sciences for their constant support.
